# Understanding the prevalence of SARS-CoV-2 (COVID-19) exposure in companion, captive, wild, and farmed animals

**DOI:** 10.1080/21505594.2021.1996519

**Published:** 2021-12-10

**Authors:** Hannah L. Murphy, Hinh Ly

**Affiliations:** Department of Veterinary & Biomedical Sciences, Comparative & Molecular Biosciences Graduate Program, College of Veterinary Medicine, University of Minnesota, Twin Cities, MN, USA

**Keywords:** COVID-19, SARS-CoV-2, pets, cat, dog, mink, deer, seroprevalence, Elisa, zoonoses

## Abstract

Several animal species, including ferrets, hamsters, monkeys, and raccoon dogs, have been shown to be susceptible to experimental infection by the human severe acute respiratory syndrome coronaviruses, such as SARS-CoV and SARS-CoV-2, which were responsible for the 2003 SARS outbreak and the 2019 coronavirus disease (COVID-19) pandemic, respectively. Emerging studies have shown that SARS-CoV-2 natural infection of pet dogs and cats is also possible, but its prevalence is not fully understood. Experimentally, it has been demonstrated that SARS-CoV-2 replicates more efficiently in cats than in dogs and that cats can transmit the virus through aerosols. With approximately 470 million pet dogs and 370 million pet cats cohabitating with their human owners worldwide, the finding of natural SARS-CoV-2 infection in these household pets has important implications for potential zoonotic transmission events during the COVID-19 pandemic as well as future SARS-related outbreaks. Here, we describe some of the ongoing worldwide surveillance efforts to assess the prevalence of SARS-CoV-2 exposure in companion, captive, wild, and farmed animals, as well as provide some perspectives on these efforts including the intra- and inter-species coronavirus transmissions, evolution, and their implications on the human-animal interface along with public health. Some ongoing efforts to develop and implement a new COVID-19 vaccine for animals are also discussed. Surveillance initiatives to track SARS-CoV-2 exposures in animals are necessary to accurately determine their impact on veterinary and human health, as well as define potential reservoir sources of the virus and its evolutionary and transmission dynamics.

## Introduction

**Figure 1.**
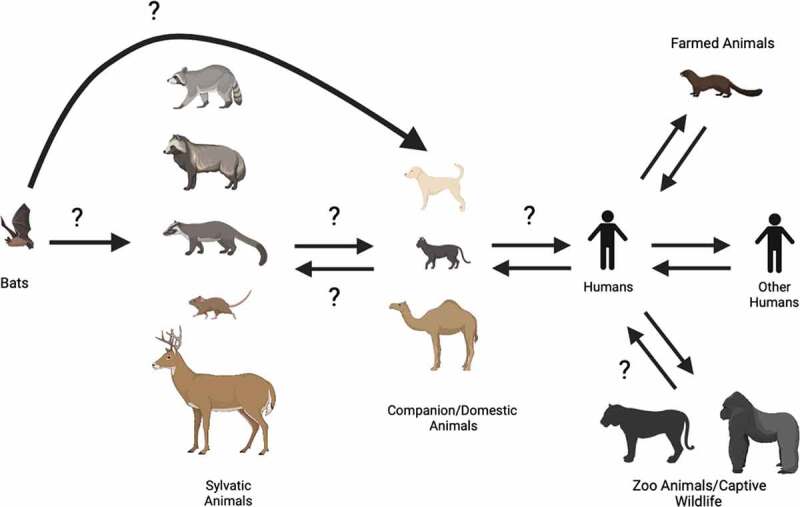
Transmission cycle of coronaviruses. Coronaviruses, including severe acute respiratory syndrome coronavirus (SARS-CoV), Middle Eastern respiratory syndrome virus (MERS-CoV), and SARS-CoV-2, are thought to originate from bats and transmit through a variety of intermediate hosts such as civet cats (in the case of SARS-CoV), camels (MERS-CoV), and other unknown species (SARS-CoV-2). At the beginning of the SARS-CoV-2 pandemic it was thought that companion animals, like cats and dogs, were not susceptible to the virus, however, this quickly changed starting in February 2020 when the first cases of human-to-animal transmission were recorded in Hong Kong. Additionally, other animal species have been shown to be exposed to SARS-CoV-2, including farmed animals (e.g., mink), sylvatic animals (e.g., white tailed deer and deer mice), as well as zoo animals (e.g., big cats and primates)

The novel 2019 coronavirus disease (COVID-19) that quickly swept the globe has caused over 220 million confirmed cases and over 4.5 million associated deaths from late 2019 to September 2021[1]. The causative agent of COVID-19 is the severe acute respiratory syndrome coronavirus 2 (SARS-CoV-2). SARS-CoV-2 is a betacoronavirus closely related to SARS-CoV and the Middle Eastern respiratory syndrome virus (MERS-CoV), which caused the 2003 and 2012 respiratory illness outbreaks. These coronaviruses are known or thought to originate from bats and transmit to humans through intermediate hosts, such as the civet cats (SARS-CoV), camels (MERS-CoV), and other yet unknown species (SARS-CoV-2) (Figure 1). SARS-CoV-2 shares 96% sequence identity to RaTG13, which is a SARS-CoV-like coronavirus isolated from the *Rhinolophus affinis* bat [2–8]. These betacoronaviruses, including SARS-CoV-2, are positive-sense single-stranded RNA viruses whose genomes encode four structural proteins [spike (S), envelope (E), membrane (M), and nucleocapsid (N)] and 16 non-structural proteins[9]. The S protein is comprised of two subunits S1 and S2. The S1 subunit contains the receptor-binding domain (RBD), which engages with the host cellular receptor [e.g., human angiotensin-converting enzyme 2 (hACE2) for SARS-CoV and SARS-CoV-2] to allow for viral entry into the host cell[10].

Many animal species are known to be susceptible to experimental infection of SARS-CoV or SARS-CoV-2, including ferrets, Syrian hamsters, deer mice, white-tailed deer, cynomolgus macaques, rhesus macaques, crab-eating macaques, African green monkeys, baboons, raccoon dogs and many others [11,12] (for a complete list, see [13]). At the beginning of the COVID-19 pandemic, it was thought that household or pet dogs and cats were not susceptible to SARS-CoV-2. However, this changed in February 2020 when the first cases of human-to-animal transmission of SARS-CoV-2 was recorded in Hong Kong, where an elderly Pomeranian dog with numerous preexisting health conditions and a 2.5-year-old male German shepherd were tested positive for SARS-CoV-2 via reverse transcription polymerase chain reaction (RT-PCR) and serology [14]. Viral genetic sequences from these two dogs were found to be identical to the virus detected in the respective human owners, supporting the role of reverse zoonotic infection. Since then, other cases of SARS-CoV-2 natural infections of both pet dogs and cats have been reported [15–18], but the extent of natural infections in companion animals is not fully understood. This mini-review attempts to provide a succinct summary of recent surveys of SARS-CoV-2 exposure in companion animals as well as in other animal species, the different techniques (e.g., serological vs. genomic) used in the analyses (Table 1), and to provide some general perspectives on these studies and their implications in reverse zoonotic viral transmissions of SARS-CoV-2. The findings of natural SARS-CoV-2 infection in household pets and other farmed, wild, and captive animals have important implications for potential zoonotic transmission events during the COVID-19 pandemic as well as in future coronavirus outbreaks.

**Table 1.** Summary of cited studies

Species of animal	Location	Clinical symptoms (present or not)	Inoculation route	Seroprevalence	Methods used for detection and/ or verification	Citation number
Cats and Dogs	Minnesota (surrounding states), USA	N/A	N/A – Pets	Cats: 11–12% May and June 2020. 5% April 2020. Dogs: 1% April to June 2020	ELISA-N protein and RBD SARS-CoV-2 spike-pseudotyped VSV assay	[19]
Cats	Wuhan, China	N/A	N/A – Pets	~15% January to March 2020.	ELISA – N and S proteins Western Blot	[20]
Cats and Dogs	Texas, USA	N/A	N/A – Pets	June to July 2020 Cats: 17.6% (3/17) Dogs: 1.7% (1/59)	RT-PCR	[18]
Cats and Dogs	Northern Italy	N/A	N/A – Pets	Cats: 5.8% Dogs: 3.3%	Plaque reduction neutralization test and RT-PCR (no positives via RT-PCR)	[23]
Cats and Dogs	France	N/A	N/A – Pets	0%	LIPS and RT-PCR	[24]
Cats	China	N/A	N/A – Pets	12% (6/50)	RT-PCR	[25]
White-tailed deer	Illinois, Michigan, New York, and Pennsylvania	N/A	N/A – Wild Animals	33% (159/481)	ELISA	[50]
Wild boar, red fox, and Jackals	Croatia	N/A	N/A – Wild Animals	Overall: 2.8% (15/533) Boar: 3.9% (6/153) Fox: 2.9% (6/204) Jackal: 4.6% (3/65)	RT-PCR and ELISA	[53]

### Serological surveys of SARS-CoV-2 exposure in companion cats and dogs

A recent study published in this journal (Virulence) by Dileepan and colleagues aims to determine the seroprevalence of SARS-CoV-2-specific antibodies (i.e., the prevalence of pet dogs and cats who have SARS-CoV-2 antibodies in their blood that show they have been exposed to this virus) in dogs and cats in Minnesota (MN) [19]. To investigate the SARS-CoV-2 seroprevalence in Minnesotan pets, they developed indirect enzyme-linked immunosorbent assays (ELISAs) that are based on either the recombinant SARS-CoV-2 N protein (N-based ELISA) or the RBD of the viral spike protein (RBD-based ELISA) to screen 239 samples of cat serum, which is the protein-rich fluid of the blood that contain antibodies, and 510 dog serum samples that were collected during routine hospital visits at the Veterinary Medical Center of the University of Minnesota during the early phase of the human’s COVID-19 pandemic from mid-April to early June of 2020. They found that the seroprevalence of SARS-CoV-2 in pet cats was higher (11–12%) in May and June of 2020 than in April of 2020 (~5%). On the contrary, they found that the SARS-CoV-2 seroprevalence in pet dogs from late April to early June of 2020 remained relatively low (~1%), which is consistent with the laboratory finding that SARS-CoV-2 replicates poorly in dogs in comparison to cats [11,12]. To confirm these findings, the authors employed another assay that assesses the ability of antibodies present in the infected cat’s serum to neutralize (or inactivate) a modeled virus called SARS-CoV-2 spike-pseudotyped vesicular stomatitis virus (VSV). In other words, this assay allows the investigators to determine the capability of a particular type of antibody, which are known as neutralizing antibodies (nAbs), present in the infected cat’s serum to inhibit this modeled virus from infecting human cells under laboratory conditions. This assay indeed confirms not only the presence but also the capability of these particular nAbs in the cat’s serum to disable SARS-CoV-2 virus-like particles from infecting human cells that contain the proper viral receptor (i.e., ACE2 protein) on their surface.

It is noteworthy that a similar study conducted in Wuhan, China, which was the epicenter of the COVID-19 pandemic, employed a similar indirect ELISA to show ~15% seroprevalence in domestic cats early during the viral outbreak [20]. In addition to screening pet serum samples by ELISA for nAbs against SARS-CoV-2, the investigators confirmed their findings by performing a Western blot assay to show that some of those antibodies can react (or bind) to the viral N and S proteins embedded on a cellulose membrane. It is important to note that the study by Dileepan and colleagues [19] shows that the N-based ELISA is a much more sensitive method than the RBD-based ELISA to detect seropositive serum samples. Additionally, the same group showed in another study that two types of recombinant SARS-CoV-2 N can be purified from bacterial cells, one that is strongly associated with bacterial RNAs and another that is completely devoid of RNAs [21]. They showed that although both forms of N can be used to develop ELISAs for SARS-CoV-2 feline seropositive serum samples, that the RNA-free form of N exhibits a higher level of sensitivity than RNA-bound form [21]. As such, it is possible that by employing only the RBD-based ELISA and not using an RNA-free N based ELISA, Zhang et al [20] could theoretically have missed many of the SARS-CoV-2 exposed cats in Wuhan, China. Interestingly, 45% (47/102) of cats used in the Zhang study [20] were abandoned (stray) cats, which raises an interesting question of the origin of their infections, perhaps pointing to the potential inter-feline (i.e., intra-species) transmission events of SAR-CoV-2 in feral Wuhan cat populations.

Unlike the Zhang study [20], where samples were collected from January to March of 2020, during the predicted peak of infections (late February 2020 in Wuhan, China), Dileepan et al [19] tested serum samples from the earlier time points of the pandemic in MN, USA. It will be important to conduct a follow-up study to assess the seroprevalence of pet cats in MN during or near the peak of SARS-CoV-2 human infections (November–December 2020) [2,19,22]. Additionally, other than the discarded serum from pets, no serum samples or clinical information about the human owners were made available to the investigators. Therefore, no direct contact tracing information was available to ascertain human-to-pet transmission events. Lastly, it was not entirely clear from where those pets were coming and whether they were all indoor pets. Many of the pets were presumably coming from households in the Twin Cities area of MN but some of them might potentially come from across the Midwestern parts of the USA, and that some of them might not be strictly home bound.

Unlike the study by Dileepan et al [19] that did not have any information on the pet’s owners, another study looking at companion animals living in confirmed COVID-19 patient’s homes in Texas from June 24 to July 31 of 2020 found that cats and dogs were found to be 17.6% (3/17) and 1.7% (1/59) seropositive for SARS-CoV-2 [18]. Yet another larger scale SARS-CoV-2 serological screening of 919 companion animals in Northern Italy found 5.8% of cats and 3.3% of dogs to possess measurable SARS-CoV-2 nAb titers [23]. While different rates of seroprevalence in household pets have been found in these studies, the reason and significance of which are unknown, they appear to show that cats are more susceptible than dogs, which is similar to the finding reported by Dileepan and colleagues [19].

While household pets have been shown to be susceptible to SARS-CoV-2, their role in the virus transmission chain is unclear at this point. To address this important issue, Temmam et al [24] have recently examined the potential transmission of SARS-CoV-2 among domestic cats and dogs living in close contact with a cluster of known SARS-CoV-2 infected French veterinary students. Many of the students were living in a university residence, where their pets had ample opportunities to interact with each other and with their human owners when visiting commonly trafficked areas during daily outdoor activities. In this study, samples were taken from a biobank to create a pet’s pre-epidemic group that contains sera collected from October 2015 to October 2018, and the so-called per-epidemic group that contains sera of the veterinary student’s pets collected on March 25^th^, 2019, which was one month after the human index case was noted. The authors did not find any SARS-CoV-2 specific antibodies in either of the pet’s cohorts via the luciferase immunoprecipitation system (LIPS) assay or by RT-PCR. The LIPS assay is based on the interaction between the viral antigens, which are fused to nanoluciferase, and their corresponding antibodies in the patient’s sera and measuring the bioluminescent substrate emitted as a result of the nanoluciferase enzymatic activity when the two proteins interact. Essentially, there was no statistical difference in the laboratory results between the two cohorts of pets. Despite having intra-species contacts daily on campus ground and frequent close contacts with COVID-19 confirmed patients in small dorm rooms, there was no evidence of SARS-CoV-2 infection of any of the student’s pets. Based on these results, the authors conclude that the rate of SARS-CoV-2 transmissions between humans and their pets living in close quarters is relatively low [24]. A possible explanation for the relatively low rate of reverse zoonosis observed between the pet’s owners and their pets in this study was partly due to the potentially low levels of viral load in the infected veterinary students, which could translate to the lower rate of the virus transmissions from the infected pet’s owners to their pets than what had been previously reported [14,18,25].

### Genomic and phylogenetic analyses

Other studies have used genomic and genetics information to attempt to address the prevalence of SARS-CoV-2 exposure in pets. One study from Hong Kong, China showed that 12% of pet cats (6/50) collected in COVID-19 positive households from February 11 to 11 August 2020 were RT-PCR positive for SARS-CoV-2 [25]. In a particular case, a household had three confirmed human cases of SARS-CoV-2 infection with symptoms that started on 20 March 2029, and 30 of 2020. On March 30, the veterinarian examined their domestic shorthair cat and found it to be clinically healthy. However, swab specimens from the cat were SARS-CoV-2 positive. The genome sequence of SARS-CoV-2 that was identified in the cat was found to be nearly (99.8%) identical to the virus derived from the human owners [25]. The timeline of viral infection of this cat and its near-identical SARS-CoV-2 genome sequence from that of the human owners in the same household provide strong evidence for a human-to-animal transmission event [25].

Other researchers have also used SARS-CoV-2 phylogenetic analyses to infer intra-species (inter-feline) transmissions. For example, in a pre-print, Bashor and colleagues intranasally inoculated cats and dogs with USA-WA1/2020 isolate to compare proportions of different SARS-CoV-2 variant appearance, including insertions and deletions. For this, oropharyngeal and nasal swabs were collected from the animals up to 10 days after virus inoculation. Collected samples were sequenced and analyzed via a modern tiled amplicon technique [26] that allows for accurately determining single nucleotide and structural sequence variations in the viral populations. The results showed that viral sequences derived from cats, that were exposed via direct contact with other infected cats, had a high level of viral sequence similarity showing that novel viral mutations could be horizontally transmitted between cats [26]. Cats also had fewer consensus viral sequences (i.e., less of the same virus sequences) and shared sequence variations as compared to those in the infected dogs. Having fewer consensus virus sequences and shared virus sequence variants suggests that the virus is better adapted to cats, which is quite like virus sequence dynamics observed in human SARS-CoV-2 infections. Also, like in the case of human infections, infected dogs have lower virus titers than infected cats, which might also contribute to the viral sequence evolution dynamics in these animal species. Braun et al. used multiplex PCR to amplify the SARS-CoV-2 genomes for sequencing [27]. In this study, three domestic specific-pathogen free cats were inoculated with a second-passage SARS-CoV-2 human isolate (hCoV-19/Japan/UT-NCGM02/2020) from Tokyo. The infectious viral titers and RNA burden were calculated in all nasal swabs collected from inoculated cats. Sequencing libraries were prepared using TrueSeq Illumina library preparation and analyzed using the SARSquencer workflow. This study was performed in order to explore the genetic diversity and transmission bottleneck size of SARS-CoV-2. Transmission bottlenecks occur in pathogen populations when only a few individual pathogens are transmitted from one infected host to another to initiate a new infection, which can dramatically affect the evolution of virulence in RNA viruses [28]. From this cat transmission model, the authors found that cats have a narrow transmission bottleneck (i.e., a dramatic reduction in viral population size at the time of transmission) where fewer than 10 viruses were founding the new infections [27]. Collectively, these studies suggest that the SARS-CoV-2 virus is likely adapting to its host (i.e., cats) quickly and as early as the first course of the infection and that repeated inter-feline transmission of the virus presents the potential for acceleration of virus evolution and opportunity for a novel virus strain to emerge naturally [26,27,29].

### “Breakthrough” and intra-species transmissions

It is important to ask whether pets can be super-infected by SARS-CoV-2, a condition that is akin to the so-called breakthrough infection in some known human cases [30–32]. To address this, Gaudreault et al [33] attempted to experimentally re-infect cats with SARS-CoV-2 at 21 days post-primary virus infection. The twice-infected animals had nAbs in their blood but had lower levels of viral RNA in their tissues than after they were first infected. These results suggest that cats can be re-infected but that they can be at least partially protected from another round of infection by either the same or genetically very similar virus. Interestingly, the re-infected cats did not appear to shed virus at sufficient levels to infect co-housed, naïve animals [33]. Similarly, Bosco-Lauth and colleagues [34] used cats and dogs to study re-infection and transmission of SARS-CoV-2. Using the SARS-CoV-2 isolate WA1/2020WY96, cats were inoculated with 3 × 10^5^ plaque-forming unit (PFU, which is a measure of the number of infectious virus particles) and then 28 days later were reinoculated with the same amount of the virus. The authors observed protective immunity against SARS-CoV-2 infection in cats following repeated virus exposure. Specifically, the experimentally infected cats developed strong levels of nAbs that conferred resistant to reinfection following a second virus challenge. Infected cats had a relatively short incubation period (5 days) and virus shedding period and were found to be resistant to a virus challenge, whereas the infected dogs (at 1.4 × 10^5^ PFU) did not shed any detectable amount of the virus even though they were found to be seroconverted and developed nAb responses against the virus. Based on these findings, the authors conclude that cats and dogs are at a relatively low risk of contributing to the transmission of SARS-CoV-2, especially for indoor pets that have limited access to other humans or animals [34].

As previously mentioned, the potential risk for intra-species (cat-to-cat) transmissibility of SARS-CoV-2 is another area of importance. Bao and colleagues conducted a study to examine the susceptibility and pathogenesis of SARS-CoV-2 in domestic cats by evaluating transmissibility after intranasal inoculation with 1 × 10^6^ 50% tissue culture infectious dose (TCID_50_) [29]. The TCID_50_ assay is a method used to quantify viral titers by determining the concentration at which 50% of the virus infected cell culture display cytopathic effect. Cats housed in pairs (i.e., an infected cat as the so-called “donor” cat with a naïve “recipient” cat) underwent four natural serial passage experiments, in which the “recipient” cat was housed with the infected “donor” cat for two days, then the “recipient” cat was used as a new “donor” cat to co-house with a new “recipient” cat, etc. The results showed that in passage 1 (P1), viral RNA from throat swabs showed shorter virus shedding periods (3–11 days post inoculation) and virus shedding peaks than those of passage 0 (P0). In contrast, passage 2 (P2) and 3 (P3) animals had no viral shedding. These data suggest that virus shedding’s peak and duration significantly decreased after natural serial passaging of the SARS-CoV-2 virus among cats, even after only one round of serial passage [29]. Viral sequencing of all P1 animals showed that changes in virus sequences could not explain the attenuated transmissibility between the “donor” and “recipient” cats, as there were no changes to the viral sequences. Instead, the authors attributed the attenuated phenotype to natural variations of amino acids in the feline ACE2 receptor, and thus, reducing its binding affinity to SARS-CoV-2 spike protein, in particularly its RBD [29], which appears to be consistent with previously reported experimental results [35].

### SARS-CoV-2 exposures in captive, farmed, and wild animals

In addition to household pets, animals living at zoos, including large cats and great apes have been found to be susceptible and infected by SARS-CoV-2. One of the earliest reports of an animal living in a zoo being infected with SARS-CoV-2 came from the Bronx Zoo when a tiger contracted the virus from interactions with a zoo employee that was actively shedding virus [36]. However, six African lions, two Amus tigers, and a Sumatran tiger have also tested positive for SARS-CoV-2, presumptively from an asymptomatic zoo keeper [37]. Great apes have also shown susceptibility to COVID-19 transmission from their handlers. Great apes are highly susceptible to many pathogens of humans [38,39]. In fact, respiratory diseases of human origin have been a significant and consistently growing threat to wild populations of great apes in Sub-Saharan Africa [40]. In January 2021, the captive troop of western lowland gorillas at the San Diego Zoo Wildlife Alliance were positively diagnosed with SARS-CoV-2 infections by PCR analysis [41]. Signs and symptoms were mostly mild and consisted of coughing and nasal discharge, however, one of the gorillas with a preexisting heart condition developed pneumonia but was able to recover treatment [42].

The infection of wild gorillas and other great apes could be more serious since they are subject to coinfections and other physiological stressors that captive apes are not known to experience. Vaccines are being developed and tested in zoo animals with gorillas, bonobos, orangutans, chimpanzees, hyenas, mountain lions, bears, ferrets, and minks have already been vaccinated, and there is already discussion of the COVID-19 vaccine being used during the rehabilitation of wild animals or in free-living habituated animals [42,43].

It is still unclear whether these animals, captive or not, could be re-infected after primary virus exposure and their clinical outcomes. However, it is known that SARS-CoV-2 can evolve independently in these potential reservoir hosts, like the mink, with viral spike protein mutations that are less readily neutralized by antibodies of SARS-CoV-2 infected humans [44]. Indeed, the first known case of animal-to-human transmissions of SARS-CoV-2 occurred in Denmark, where two workers on a mink farm contracted the virus, and the virus subsequently appeared in the local human population [45]. When viral evolution can continue in potential reservoir animal hosts, like the mink and perhaps other animal species, spillback events into humans (i.e., zoonotic infections) have the potential to cause significant risk to public health [13,46–49].

Interestingly, wildlife species, such as white-tailed deer, have recently been shown to be among the list of animals that are susceptible to SARS-CoV-2 infection. The Animal and Plant Health Inspection Services (APHIS) of the U.S. Department of Agriculture (USDA) conducted a study which analyzed serum samples of free-ranging white-tailed deer for SARS-CoV-2 antibodies [50]. The results of the study showed that white-tailed deer populations in Illinois, Michigan, New York, and Pennsylvania have been exposed to SARS-CoV-2 [50]. They collected samples between January 2020 and March 2021 and detected 33% of anti-SARS-CoV-2 antibodies in the samples [50]. Although it is not known how deer were exposed to SARS-CoV-2 (e.g., through the environment, people, or other animals), this new piece of the data highlights another potential source of wild animals that can be infected by SARS-CoV-2.

Peromyscine rodents in the genus *Peromyscus* include 56 species that are distributed throughout North America. Many rodents serve as reservoir hosts for a diverse array of zoonotic agents including hantaviruses, *Borrelia burgdorferi* (a causative agent of Lyme disease), *Babesia* species, and Powassan virus. Rodent habitats are often near farmed animal enclosures or even inside the enclosures themselves alongside the farmed animals. Since there is a close interaction between rodents and livestock, it is not hard to imagine that there is a farm and wild animal interface where inter-species transmission of SARS-CoV-2 is possible. Although seroprevalence studies have not been done on wild deer mice yet, adult deer mice have been experimentally infected in the laboratory with SARS-CoV-2 with mixed results of susceptibility to severe disease [51,52].

In one such studies, deer mice were intranasally challenged with 2 × 10^4^ TCID_50_ of SARS-CoV-2. The infected animals showed substantial pulmonary consolidation and hemorrhage in the cranial and middle portions of the lungs [52]. These pathological findings demonstrate that deer mice are susceptible to SARS-CoV-2 infection but also could be used as a suitable small animal model for studying SARS disease pathology since they exhibit similar clinical presentations to humans [52]. However, more studies are needed to confirm these results since another laboratory study, in contrast, found that deer mice, when intranasally inoculated with 1 × 10^5^ TCID_50_ of SARS-CoV-2, were either asymptomatic or exhibited only a relatively mild form of the disease, despite having high viral titers and elevated inflammatory cytokines in the lungs [51]. In another study, the investigators looked for the presence of SARS-CoV-2 antibodies and viral RNA in 422 samples collected from free-living and captive wildlife species in Croatia from June 2020 to February 2021 and found 2.8% of the 422 samples were positive, among which 3.9% wild boars, 2.9% red foxes, and 4.6% jackals were found to be positive, despite no obvious signs of natural infection [53].

### SARS-CoV-2 vaccine for animals

A possible solution to halt viral evolution in the reservoir animals and spillback events to infect humans could be through vaccination of animals that are known to be susceptible to SARS-CoV-2 infection [49]. Toward this front, Zoetis, which is a major U.S. veterinary pharmaceutical company, has already made some progress in developing a SARS-CoV-2 vaccine for animals. It has recently conducted preliminary studies for a dog and cat vaccine that functions similarly to the Novavax’s subunit vaccine in humans. Specifically, it uses a synthetic nanoparticle to display SARS-CoV-2 spike proteins to the vaccinated individual, which has shown to be safe and relatively effective [54]. Since mink culling in Europe, Zoetis has shifted its focus toward a mink vaccine as Denmark and other fur-producing countries have demonstrated an urgent need for such a vaccine [54]. Interestingly, dogs, cats, and mink are not the only species of animals that would benefit from vaccination, great apes at the San Diego Zoo have also recently been vaccinated with the COVID-19 vaccine. Similarly, other vulnerable species, including gorillas, chimpanzees, and orangutans at the San Diego Zoo, were vaccinated with the Zoetis COVID-19 vaccine in February 2020 [43,55].

## Concluding remarks

It is well established that human-to-human transmission is the main route for SARS-CoV-2 circulation in the world’s human populations. However, it is becoming increasingly evident that the virus can also infect and replicate (multiply) in various animal hosts, including companion, captive, wild, and farmed animals. Therefore, surveillance to track virus prevalence in wild and domesticated animals is urgently needed. This will allow for accurate determination of the impact that animals have had and will continue to have as potential reservoir hosts for SARS-CoV-2 and their impact on human transmission dynamics. The continued development and improvement of SARS-CoV-2 detection assays are needed to maintain an effective surveillance system across human and animal species. Along with active surveillance, there is a demand for a plan to control viral spread and evolution, and therefore progress of SARS-CoV-2 vaccine development for vulnerable animals needs to be encouraged to keep viral transmissions under control in all susceptible hosts, regardless of the species.

## Data Availability

No primary data (figures and tables) are included in this article. Regardless, an original (preprint) version of this article has been deposited in a recognized data repository (Figshare.com) with a digital object identifier 10.6084/m9.figshare.16591028
